# Cholera, the Current Status of Cholera Vaccines and Recommendations for Travellers

**DOI:** 10.3390/vaccines8040606

**Published:** 2020-10-14

**Authors:** Giovanni Gabutti, Andrea Rossanese, Alberto Tomasi, Sandro Giuffrida, Vincenzo Nicosia, Juan Barriga, Caterina Florescu, Federica Sandri, Armando Stefanati

**Affiliations:** 1 Department of Medical Sciences, University of Ferrara, 44121 Ferrara, Italy; armando.stefanati@unife.it; 2 Department of Infectious Tropical Diseases and Microbiology, IRCCS “Sacro Cuore-Don Calabria”, Negrar di Valpolicella, 37024 Verona, Italy; andrea.rossanese@gmail.com; 3 Italian Society of Travel and Migration Medicine (SIMVIM), 00185 Rome, Italy; a.tomasi224@gmail.com; 4 Local Health Unit, Department of Prevention, 89121 Reggio Calabria, Italy; sandrogiuffrida@gmail.com; 5 Head of Health and Occupational Medicine Saipem SpA, 20097 Milan, Italy; vincenzo.nicosia@saipem.com; 6 Department of Medical Affairs Europe, Emergent BioSolutions, 1455 Madrid, Spain; barrigaj@ebsi.com; 7 Postgraduate School of Hygiene and Preventive Medicine, University of Ferrara, 44121 Ferrara, Italy; flrcrn@unife.it (C.F.); sndfrc@unife.it (F.S.)

**Keywords:** cholera, cholera prevention, vaccines, oral cholera vaccine

## Abstract

Cholera is endemic in approximately 50 countries, primarily in Africa and South and Southeast Asia, and in these areas, it remains a disease associated with poverty. In developed nations, cholera is rare, and cases are typically imported from endemic areas by returning travellers. Cholera is readily preventable with the tools available to modern medicine. In developing nations, cholera transmission can be prevented through improved water, sanitation, and hygiene services and the use of oral cholera vaccines (OCVs). For travellers, risk can be mitigated by practicing regular hand hygiene and consuming food and water from safe sources. OCVs should be considered for high-risk travellers likely to be exposed to cholera patients or contaminated water and food. There are currently three World Health Organization pre-qualified OCVs, which are based on killed whole-cell strains of *Vibrio cholerae*. These established vaccines offer significant protection in adults and children for up to 2 years. A novel live attenuated vaccine that provides rapid-onset protection in adults and children is licensed in the USA and Europe only. Live attenuated OCVs may mimic the natural infection of *V. cholerae* more closely, generating rapid immune responses without the need for repeat dosing. These potential benefits have prompted the ongoing development of several additional live attenuated vaccines. The objective of this article is to provide a general review of the current landscape of OCVs, including a discussion of their appropriate use in international travellers.

## 1. Cause and Symptoms of Cholera

Cholera is an acute, secretory diarrhoeal disease caused by toxigenic strains of the Gram-negative bacterium *Vibrio cholerae*. There are over 200 serogroups of *V. cholerae*, which are distinguished based on the O antigen of surface lipopolysaccharides, although only two are capable of secreting cholera toxin (CT) and causing disease [1,2]. The O1 serogroup is subdivided into “classical” and “El Tor” biotypes, and the genes for CT, contained within the genome of the bacteriophage CTXφ, differ between these two biotypes [2]. A new disease-causing serotype, O139, which emerged throughout Asia in 1992, is derived from O1 El Tor organisms via horizontal gene transfer. However, El Tor currently accounts for almost all worldwide cases of cholera, and O139 is seldom isolated [1]. Some El Tor strains may cause a more severe form of the disease due to the increased secretion of CT [3].

Infection occurs orally, via the ingestion of contaminated water and/or food [4], and organisms that survive the gastric environment colonise the small intestine. The virulence of *V. cholerae* is determined by two critical factors: CT and toxin coregulated pilus (TCP). These factors are under the control of a signalling cascade known as the ToxR regulon, which responds to environmental stimuli to regulate virulence factor production in the host environment [5]. TCP is fundamental for colonisation and facilitates the aggregation of vibrios into colonies that protect them from host immune responses [6], whereas CT is essential for enterotoxicity [5].

**Figure 1:**
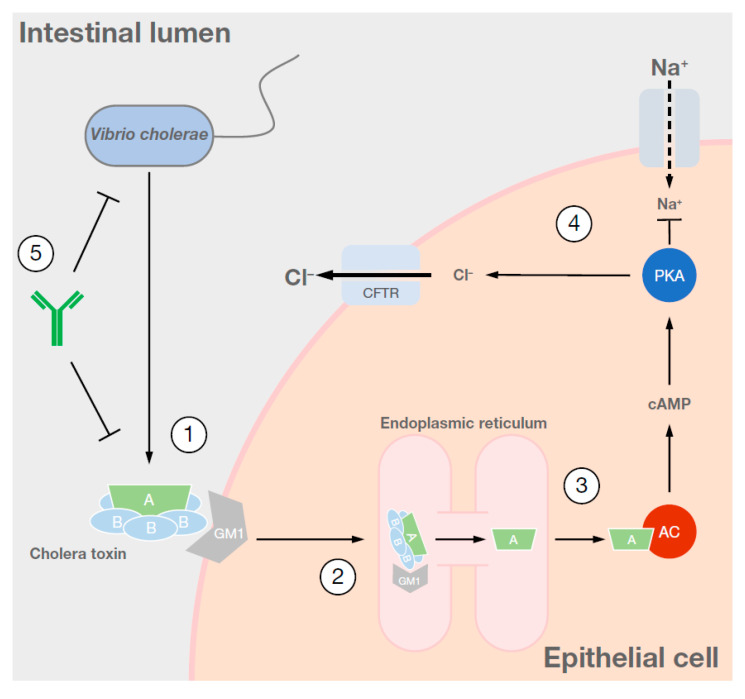
Cholera toxin mechanism of action. (**1**) Upon ingestion, *Vibrio cholerae* colonises the intestine and produces cholera toxin, consisting of the toxic A subunit and five B subunits, which are responsible for receptor binding. (**2**) Once bound to GM1 receptors, cholera toxin is endocytosed and trafficked to the endoplasmic reticulum where the A and B subunits disassociate. (**3**) The A subunit stimulates the adenylate cyclase complex, resulting in an increase in intracellular cAMP. (**4**) cAMP activates protein kinase A which, in turn, phosphorylates the CFTR chloride channel, resulting in an efflux of chloride ions from the cell and a reduction in uptake of sodium ions. The resulting osmotic gradient leads to an overall movement of water into the intestinal lumen, resulting in the secretory diarrhoea characteristic of cholera. (**5**) Cholera vaccines promote the generation of antibodies against cholera toxin, which block receptor binding, or against cholera vibrios, which immobilise the pathogen and prevent colonisation. A, cholera toxic A subunit; AC, adenylate cyclase; B, cholera toxic B subunit; cAMP, cyclic adenosine monophosphate; CFTR, cystic fibrosis transmembrane conductance regulator; Cl, chloride ion; GM1, monosialotetrahexosylganglioside receptor; Na, sodium ion; PKA, protein kinase A.

CT binds to GM1 ganglioside receptors on the surface of intestinal epithelial cells and is translocated intracellularly via endocytosis. The toxin is processed in the endoplasmic reticulum and the active subunit stimulates cyclic AMP production via the adenylate cyclase complex. The subsequent activation of kinases triggers an efflux of chloride ions into the intestinal lumen and inhibits the uptake of sodium ions (Figure 1) [1,2]. The resulting osmotic potential draws out water from the surrounding tissues, resulting in the predominant symptom of diarrhoea [4]. Other symptoms include vomiting, circulatory collapse, and shock, although many infections are associated with milder diarrhoea or have no symptoms at all [7].

It takes between 12 h and 5 days for a person to develop symptoms after consuming contaminated food or water [4]. Approximately 10% of infected persons will have severe disease, which is characterised by profuse, watery diarrhoea, often described as “rice-water stool” owing to the pale, milky appearance [7,8]. Susceptibility to infection depends on immune responses, triggered by previous exposure to *V. cholerae* by either infection or vaccination, and on innate host factors.

If left untreated, 25–50% of severe cholera cases can be fatal [7]. Although 90–95% of infected people will remain asymptomatic or experience only mild symptoms [9], *V. cholerae* can remain present in their faeces for 1–10 days following infection, causing the bacteria to be shed back into the environment, increasing the risk of further infections [4].

## 2. Epidemiology of Cholera

### 2.1. Global Overview

Cholera may be either endemic or epidemic, with endemic cholera representing most cases [4,10]. An area or country is considered cholera-endemic when cholera cases have been detected during the last 3 years with evidence of local transmission rather than cases imported from elsewhere [4]. A cholera outbreak can occur in areas where cholera is already endemic when the number of observed cases is greater than expected [4]. Outbreaks in countries where cholera does not frequently occur are defined by the occurrence of at least one confirmed case of cholera with evidence of local transmission [4]. Epidemics of cholera are difficult to predict; they may be seasonal (e.g., increasing during flood seasons), but they can also be sporadic [4].

Cholera is endemic in approximately 50 countries [8,11], primarily in South Asia, Southeast Asia, and Africa [8,12], but this number is variable as the countries affected, according to the Centers for Disease Control and Prevention (CDC) website, change frequently [7]. Additionally, it is estimated that only a small proportion of cholera cases are officially reported [10]. Nevertheless, the global burden of cholera is estimated to be between 1.3 and 4.0 million cases, with about 21,000–143,000 deaths per year [10]. In 2017 alone, 34 countries reported a total of 1,227,391 cases and 5654 deaths (global case-fatality rate: 0.5%) [11].

### 2.2. Cholera in Developed Countries

Cholera can be imported from areas where it is endemic or epidemic to cholera-free countries [13]. From 2010 to 2016, 107 cases of cholera were confirmed in the USA among people who had travelled internationally in the week before the onset of symptoms [8]. In 2017, 675 cases of imported cholera were reported to the WHO, with 12 of these being recorded in North America [11]. However, the number of cholera cases in the USA is estimated to be up to 33 times higher than those diagnosed [14]. In Europe during 2018, 26 confirmed cases of cholera were reported across five countries. The UK accounted for the highest number of cases (76.9%), which is consistent with observations from previous years.

### 2.3. Cholera in Travellers

Exposure to cholera infection is a risk for individuals and groups from non-endemic areas travelling to endemic countries or regions where there is an active epidemic [8]. These groups may include individuals engaging in humanitarian, medical, or missionary work, tourists, business travellers, or military personnel. Nevertheless, cholera is a rare disease among travellers from non-endemic to endemic areas, with a risk of approximately 0.2 cases per 100,000 North American and European travellers [15]. Travellers may be at higher risk if they are visiting family and friends in a region experiencing an outbreak or are working in high-risk settings (such as cholera treatment centres or refugee camps). These travellers may also be at higher risk because they stay longer or have reduced access to safe food and water [8]. In healthy adults travelling to endemic areas, cholera is rarely distinguished from, and routinely treated as, acute travellers’ diarrhoea (TD) and is under-reported as a specific cause of illness [8,13,16].

### 2.4. Under-Reporting of Cholera Cases

It is generally recognised that there may be widespread under-reporting and underdiagnosis of cholera globally due to political and social disincentives, economic pressures, lack of diligence, or inadequate investigation [10,17]. The WHO has estimated that officially reported cases represent only 5–10% of the true number [10]. The differentiation of cholera from other diarrhoeal diseases based on clinical observations is often difficult [13,18], and poor laboratory facilities and epidemiological surveillance in endemic regions also complicate accurate diagnosis. The isolation and identification of a *V. cholerae* serogroup O139 or O1 by laboratory culture of a stool sample remains the gold standard for the diagnosis of cholera [7]. In regions with limited testing facilities, rapid dipstick testing can provide early warning of an epidemic. However, the specificity and sensitivity of dipstick testing is not wholly reliable (sensitivity ≈ 90%; specificity ≈ 50%) [17].

Furthermore, within an outbreak setting, not all specimens may be tested [10,18]. Additionally, some countries may suppress reports because of perceived risks to export industries and/or tourism [19]. The WHO acknowledges that its surveillance methods provide only a partial picture of global cholera, resulting in inconsistent data, as countries with significant endemic seasonal transmission still do not publicly count or report cases of cholera [11]. 

### 2.5. Natural Immunity

*V. cholerae* induces long-lasting immunity in most people who recover from infection [20]. Studies have demonstrated that cholera infection results in 65–100% protection against reinfection for at least 3 years [20]. Research on adaptive immunity to cholera has centred primarily on antibodies, as antibody responses are considered to confer protection at the mucosal surface [21]. Antibody responses directed against cholera toxin do not appear to provide long-term protective immunity, but they do contribute to short-term protection. This is consistent with studies demonstrating that oral cholera toxin vaccine provides limited, short-term protection against severe cholera [20].

By contrast, antibacterial responses are crucial for long-term protective immunity against cholera [20]. Presently, the best measure of protection against cholera is the vibriocidal antibody titre, which measures the minimum concentration of serum required for antibody-dependent complement-mediated bacterial killing [20]. Nevertheless, vibriocidal antibodies are an imprecise marker of cholera protection, and although antibody-dependent complement-mediated bacterial killing is a prominent defence against systemic infections, it is not thought to be pertinent at the intestinal surface due to low levels of complementation at this site [22]. Instead, the immobilisation of bacterial pathogens in the mucus layer and restriction of motility are functions of antibodies that are considered likely to prevent colonisation by *V. cholera* [23,24]. This absence of a direct mechanistic connection between circulating vibriocidal antibodies and immunity may explain their limitation as an indicator of protection [22]. However, despite this limitation, the vibriocidal antibody response appears to be a convenient proxy marker for a longer-lasting mucosal immune response [20].

## 3. Prevention of Cholera in Travellers

Any people travelling to cholera-endemic areas should be apprised of the risk, understand the watery diarrhoea presentation of cholera infection, and be provided with appropriate information about self-treatment options (oral rehydration) to mitigate morbidity [25]. For anyone travelling to regions with active cholera transmission, the primary prevention strategy should involve following safe food and water precautions, proper sanitation, and good personal hygiene measures such as frequent handwashing [26]. Vaccination should be considered for travellers at high risk [26].

### 3.1. Preventive Hygiene Measures Including Washing Hands, Food Control, etc.

The risk of infection for most international travellers is extremely low, even in countries where cholera outbreaks are active, as long as appropriate preventive measures are followed [27]:

- Practice regular hygiene, especially hand hygiene with soap and water or, if not available, with an alcohol-based hand sanitiser solution;
- Practice hand hygiene especially before touching the nose, eyes, or mouth, and after using the toilet or touching objects at high risk of being contaminated;
- All precautions should be taken to avoid the ingestion of potentially contaminated food, drink, and drinking water by consuming food and water only from safe, known sources;
- Follow the five rules for food safety: wash hands often and always before handling and consuming food; make sure the food is cooked thoroughly; peel all vegetables and fruit if they are to be eaten raw; drink bottled water where available, or if the source of water is uncertain, bring it to a vigorous boil; separate cooked food from raw food, avoid uncooked food, and keep food at safe temperatures.

### 3.2. Chemoprophylaxis

Antibiotic prophylaxis for travellers arriving in or departing from cholera-affected areas has not been shown to have any effect on the spread of cholera but can have negative effects such as driving antimicrobial resistance and providing a false sense of security. Therefore, the WHO and CDC do not advise prophylactic administration of antibiotics [7,27].

### 3.3. Vaccination

Oral cholera vaccines (OCVs) should be considered for travellers at high risk who are likely to be directly exposed to cholera patients or contaminated water and food, particularly those staying in areas with limited access to health care facilities [27]. The Advisory Committee on Immunization Practices and the CDC recommend the vaccination of US travellers to areas of active cholera transmission, which are defined as a province, state, or other administrative subdivision within a country with endemic or epidemic cholera caused by toxigenic *V. cholerae* O1 [26]. In addition to considering the area of travel, several European countries, Canada, and Australia have formulated their recommendations to take into account the type of travel being undertaken, whether the individual will be undertaking any high-risk activities during their trip, and whether they have any underlying health conditions that increase the risk of developing severe disease. A summary of these recommendations is given in Table 1. 

## 4. Vaccines Available for Cholera

The first vaccine against cholera, a live whole-cell injectable vaccine, was developed in 1885 by the Spanish bacteriologist Jaume Ferran i Clua, following early observations that people who recovered from cholera were protected against subsequent infections during the same epidemic [55]. Early work developing killed culture vaccines was reported by Ukrainian physician Nikolay Gamaleya in 1888, and Waldemar Haffkine described efforts with attenuated strains in 1892 [56,57,58]. Other injectables were developed throughout the early 20th century but are not recommended by the WHO due to low levels of efficacy, short duration of protection, and unfavourable safety profiles [55,59]. The first whole-cell oral vaccine to be licensed internationally was developed at the University of Gothenburg, Sweden and pre-qualified by the WHO in 2001 [55,60]. Currently, there are three WHO pre-qualified OCVs (Table 2) [61,62,63], and a global OCV stockpile was established in 2013, with long-term support from Gavi, the Vaccine Alliance. This initiative has facilitated a large increase in the targeted use of OCVs in epidemic and humanitarian settings following leadership from the Global Task Force on Cholera Control (GTFCC). The latest available data (May 2018) show that more than 25 million doses have been administered through mass vaccination campaigns in 19 countries since the stockpile was established [64].

A fourth vaccine, previously available only in the USA [65], was approved by the European Medicines Agency, as of April 2020 [66], for immunisation against disease caused by the *V. cholerae* serogroup O1 in adults and children aged 6 years and older [67] (Table 2).

### 4.1. WC-rBS, Killed Whole-Cell Monovalent (O1) Vaccines with a Recombinant B Subunit of Cholera Toxin (Dukoral^®^)

WC-rBS was the first killed whole-cell oral vaccine to be licensed internationally (in 1991) [55], and it is available in >60 countries [12]. WC-rBS is derived from three killed strains of *V. cholerae* O1 bacteria (*V. cholerae* O1 Inaba classical [heat inactivated], *V. cholerae* O1 Inaba El Tor [formalin inactivated], and *V. cholerae* O1 Ogawa classical [heat inactivated and formalin inactivated]), and recombinant cholera toxin beta (CTB) [68]. The vaccine induces antibodies against both the bacterial components and the CTB; the bacteria-induced antibodies impede colonisation of the intestine by *V. cholerae* O1 by preventing bacterial attachment to the intestinal wall, and the antitoxin antibodies attenuate diarrhoeal symptoms by preventing the cholera toxin from binding to the intestinal mucosa [68]. Prior to 1991, the vaccine contained a native B subunit purified from cholera toxin produced by a wild-type strain, which was replaced by recombinant CTB after this date [55], with comparable immune responses and safety profile [69,70,71]. A similar vaccine, also containing a recombinant cholera toxin B subunit, *OraVacs*™, is currently only available in China and the Philippines [72].

WC-rBS is primarily aimed at travellers and is indicated for active immunisation against disease caused by *V. cholerae* O1 in adults and children from 2 years of age who will be visiting endemic areas or regions experiencing an epidemic [68]. Immunisation is recommended at least 1 week prior to potential exposure to *V. cholerae* O1, with the primary course comprising two doses in adults and children > 6 years of age, and three doses for children aged <6 years [68]. A booster vaccination is recommended within 2 years for adults and children aged >6 years of age and within 6 months for children aged 2–6 years [68]. The efficacy of WC-rBS was assessed in three randomised, double-blind clinical trials; the Bangladesh field trial [73,74], a trial involving military personnel in Peru [75], and a field trial in Peru [68,76].

In the Bangladesh field trial, protective efficacy in the overall population was 85% (95% confidence interval [CI] 56–95) during the preliminary 6 months of follow-up. However, the duration of vaccine protection fluctuated by age, lasting for 6 months in children and for 2 years in adults [68,73]. This difference in the duration of protection is reflected in the recommended dosing regimen [68]. An exploratory analysis suggested that a two-dose regimen was as effective as a three-dose regimen in adults [68].

The short-term protective efficacy in the military trial in Peru was comparable at 86% (95% CI 37–97; *p* < 0.01), with an average follow-up of 5 months [75]. By contrast, in the field trial in Peru, there was no protective effect against cholera in the first year, and only 61% (95% CI 28–79) following the booster vaccination at 1 year [76]. The vaccine was observed to be more efficacious in individuals older than 15 years of age in this study, implying that three doses may be more effective in younger children [76], which is reflected in the current dosing recommendations [68]. The lack of effect within the first year was later attributed to methodological issues [77].

Protective effectiveness against cholera was also evaluated during two mass-vaccination campaigns in Mozambique [78] and Zanzibar [79]. In the case-control study conducted in Mozambique, the protective effectiveness of two doses of WC-rBS was 84% (95% Cl 43–95, per-protocol analysis; *p* = 0.005) over the initial 5 months of follow-up [78]. In the longitudinal cohort analysis conducted in Zanzibar, the protective effectiveness provided by two doses of WC-rBS was 79% (95% Cl 47–92) over a follow-up period of 15 months [79]. In addition to the direct protection, it was demonstrated that WC-rBS provided substantial herd (indirect) protection in the observed setting [79].

### 4.2. BivWC, Killed Modified Whole-Cell Bivalent (O1 and O139) Vaccines without B Subunit

A second OCV, *Shanchol^®^*, is derived from three killed strains of *V. cholerae* O1 bacteria and one killed O139 strain but no cholera toxin B subunit [80], making administration easier, and it acts to prevent the colonisation of both *V. cholerae* O1 and O139 in the gut [81]. *Shanchol^®^* is indicated for active immunisation against *V. cholerae* in anyone aged >1 year [81]. A closely related vaccine, *mORCVAX*™, is formulated by a separate manufacturer and available only in Vietnam [55], whereas another, *Cholvax*, is currently under development for the Bangladesh market only [72]. The primary immunisation schedule consists of two doses given at an interval of 2 weeks, with the earliest onset of protection following 7–10 days after completion [81]. Protective effectiveness against cholera was also evaluated during mass-vaccination campaigns in Vietnam and India [55,80,82,83].

During an outbreak of the O1 El Tor biotype, 8–10 months following vaccination in Vietnam, 37 cholera cases that occurred among age-eligible individuals allocated to the vaccine group required hospital care, in contrast to 92 cases among age-eligible people allocated to the no-vaccine group (protective impact 60% [95% CI 40–73]). Among the 51,975 people who received the recommended two-dose vaccine regimen, the protective efficacy was 66% (95% CI 46–79) [80]. In India, from the 66,900 individuals vaccinated, 20 episodes of cholera were reported in the vaccine group compared with 68 episodes in the placebo group (protective efficacy 67%; one-tailed 99% CI, lower bound 35%, *p* < 0.0001). The vaccine provided protection to individuals in the age groups 1.0–4.9 years, 5.0–14.9 years, and 15 years and older. Protective efficacy was not significantly different between groups (*p* = 0.28) [82]. Additionally, protection during a third year of follow-up was 65% (one-sided 95% CI, lower bound 44%, *p* < 0.001) [84]. In a further trial in India, of 51,488 eligible residents, 31,552 individuals received one dose and 23,751 residents received two doses of vaccine. Protective effectiveness for persons receiving two doses was 69% (95% CI 14.5–88.8), and statistical analysis suggested that a single dose still provided a degree of protection (33%, *p* = 0.0091) [83].

A third pre-qualified OCV, *Euvichol™/Euvichol-Plus™* is a liquid formulation of an OCV containing three O1 serotypes and one O139 serotype, inactivated by heat or formalin [85]. *Euvichol™/Euvichol-Plus™* is indicated for anyone aged >1 year, with two doses to be administered orally at an interval of 2 weeks [85].

In a non-inferiority trial, conducted in a total of 1263 healthy participants (777 adults and 486 children), two doses of either *Shanchol^®^* or *Euvichol™/Euvichol-Plus™* were administered. Vibriocidal antibody responses to *V. cholerae* O1 Inaba following the administration of two doses of *Euvichol™/Euvichol-Plus™* were non-inferior to those of *Shanchol^®^* in children (87% vs. 89%) and in adults (82% vs. 76%). Comparable findings were also observed for O1 Ogawa in children (91% vs. 88%) and adults (80% vs. 74%). No serious adverse reactions were reported in either vaccine group [86]. Furthermore, a meta-analysis has indicated that the average efficacy for a two-dose regimen is 58% (95% CI 42–69) over 3 years. In those studies reporting results stratified by age, children aged 1 to <5 years old had a 30% (95% CI 15–42) vaccine efficacy [87]. 

### 4.3. Recombinant Live OCV CVD 103-HgR (Vaxchora^®^)

CVD 103-HgR, previously only available in the USA, is the sole cholera vaccine available for US travellers [65]. CVD 103-HgR is an oral, live attenuated bacterial vaccine containing *V. cholerae* strain CVD 103-HgR, which is constructed from the serogroup O1 classical Inaba strain 569B.38. It is indicated in the USA for active immunisation against disease caused by *V. cholerae* serogroup O1 in adults aged 18–64 years travelling to cholera-affected areas [65]. The approval of CVD 103-HgR in other global markets is pending. However, as of April 2020 [66], CVD 103-HgR was granted marketing authorisation in EEA after being reccomended by the European Medicines Agency for use in adults and children aged 6 years and older [67].

CVD 103-HgR is a single-dose oral vaccine that provides rapid-onset protection [65]. The live attenuated strain CVD 103-HgR is unable to synthesise active cholera toxin (the major virulence factor for *V. cholera* [2]), but it is able to synthesise the immunogenic non-toxic CTB and replicates in the gastrointestinal tract. Studies indicate a rise in vibriocidal antibody 10 days after vaccination, which is associated with protection against *V. cholerae*. Although the duration of protection has not been fully elucidated, geometric mean titres (GMTs) of serum vibriocidal antibodies in vaccine recipients were significantly higher than the corresponding GMTs of placebo subjects at 90 and 180 days [67]. Additionally, a long-term follow-up at 730 days is currently ongoing in a paediatric sub-group study [88].

In a study of 197 adult subjects, 95 received the CVD 103-HgR vaccine and 102 received placebo [65]. Sixty eight of the 95 CVD 103-HgR recipients were challenged: 35 subjects at 10 days post vaccination and 33 subjects at 3 months post vaccination. Of the 102 placebo recipients, 66 were challenged: 33 at 10 days post vaccination and 33 at 3 months post vaccination [65,89]. Of the CVD 103-HgR recipients challenged 10 days post vaccination, only two subjects (5.7%) experienced moderate or severe diarrhoea, and for those challenged 3 months post vaccination, only four subjects (12.1%) experienced moderate or severe diarrhoea. Moderate or severe diarrhoea was experienced by 39 of the 66 subjects challenged after receiving placebo (59.1%). These data indicated that CVD 103-HgR was 90.3% effective against moderate or severe diarrhoea in the 10 days post-vaccination challenge group and 79.5% effective in the 3 months post-vaccination challenge group [65,89].

Two additional studies evaluated immunogenicity, using seroconversion (four-fold or greater rise in serum vibriocidal antibody titres from baseline, measured 10 days after vaccination) as the immunologic bridge between challenge trial study and other populations [90,91]. One trial in 3146 healthy adults aged 18–45 years showed a seroconversion rate of 94% in vaccinated subjects vs. 4% in placebo recipients [91], whereas a trial in 398 healthy older adults aged 46–64 years demonstrated seroconversion rates of 90.4% in vaccinated subjects vs. no seroconversion in the placebo group [90]. Prespecified immunobridging analyses demonstrated non-inferiority in the seroconversion rate between older adults and those aged 18–45 years in a large immunogenicity trial [67]. A further immunogenicity trial, conducted in 374 healthy children aged 6 to <18 years, demonstrated that serum vibriocidal antibody seroconversion rates 10 days following immunisation were 98.6% in the vaccine group vs. 2.1% in placebo recipients. The vaccine seroconversion rate was non-inferior to a 93.5% rate seen in adults aged 18–45 years [67,88,92].

**Table 2:** Summary of currently available cholera vaccines.

Vaccine and Manufacturer	Killed Whole-Cell Monovalent (O1) Vaccine with a Recombinant Cholera Toxin B Subunit WC-rBS (*Dukoral^®^*) Valneva (Sweden) [68]	Killed Whole-Cell Bivalent (O1 and O139) Vaccine without the Cholera Toxin B Subunit BivWC (*Shanchol^®^*) Shantha Biotechnics (India) [81]	Killed Whole-Cell Bivalent (O1 and O139 without Cholera Toxin B Subunit) BivWC (*Euvichol™/Euvichol-Plus™*) EuBiologics (S Korea) [85]	Live Attenuated Vaccine CVD 103-HgR (*Vaxchora*^®^) Emergent BioSolutions (USA) [65]
Composition	*V. cholerae* O1 Inaba, classical biotype (heat inactivated) 31.25 × 10^9^ bacteria *V. cholerae* O1 Inaba, El Tor biotype (formalin inactivated) 31.25 × 10^9^ bacteria *V. cholerae* O1 Ogawa, classical biotype (heat inactivated) 31.25 × 10^9^ bacteria *V. cholerae* O1 Ogawa, classical biotype (formalin inactivated) 31.25 × 10^9^ bacteria Recombinant cholera toxin B subunit 1 mg (produced in *V. cholerae* O1 Inaba, classical biotype strain 213)	*V. cholerae* O1 Inaba El Tor strain Phil 6973 (formaldehyde inactivated) 600 LEU *V. cholerae* O1 Ogawa classical strain Cairo 50 (heat inactivated) 300 EU LEU *V. cholerae* O1 Ogawa classical strain Cairo 50 (formaldehyde inactivated) 300 LEU *V. cholerae* O1 Inaba classical strain Cairo 48 (heat inactivated) 300 LEU *V. cholerae* O139 strain 4260B (formaldehyde killed) 600 LEU	*V. cholerae* O1 Inaba Cairo 48 classical biotype (heat inactivated) 300 LEU *V. cholerae* O1 Inaba Phil 6973 El Tor biotype (formalin inactivated) 600 LEU *V. cholerae* O1 Ogawa Cairo 50 classical biotype (formalin inactivated) 300 LEU *V. cholerae* O1 Ogawa Cairo 50 classical biotype (heat inactivated) 300 LEU *V. cholerae* O139 4260B (formalin inactivated) 600 LEU	*V. cholerae* strain CVD 103-HgR (live, attenuated) 4 × 10^8^–2 × 10^9^ CFU
Pharmaceutical form	Suspension and effervescent granules for suspension	Suspension	Suspension	Suspension
Route of administration	Oral	Oral	Oral	Oral
Recommended dose/regimen	2 doses in adults and children ≥6 years of age, 3 doses for children aged <6 years; ≥1 week before potential exposure	2 doses at an interval of 2 weeks; earliest onset of protection 7–10 days after completion	2 doses at an interval of 2 weeks	Single dose; ≥10 days before potential exposure
Recommended age of vaccination	Adults and children ≥2 years of age	Adults and children ≥1 year of age	Adults and children ≥1 year of age	Adults and children aged ≥6 years
Availability	Worldwide (unavailable in the USA)	Worldwide (unavailable in the USA)	Worldwide (unavailable in the USA)	USA and Europe

CFU, colony-forming units; LEU, lipopolysaccharide ELISA Units.

## 5. Development of Live Attenuated *V. cholerae* Vaccines

Live attenuated vaccines may have reduced effectiveness in partially immune individuals residing in endemic areas, as pre-existing antibodies can impede live vibrios and reduce colonisation of the gut [72]. Furthermore, whether a live attenuated vaccine could revert to a wild-type infective strain remains unknown. However, during a clinical trial of the CVD-103 HgR vaccine, the strain was shed by only 11% of vaccine recipients, and no transmission to household contacts was observed [93]. However, live attenuated OCVs can respond to the intestinal environment and are thought to more closely mimic the natural infection process of *V. cholerae* in generating a mucosal gut immune response. Furthermore, live strains are taken up more efficiently by M cells, the major antigen sampling cells in the gut, than are killed strains [72]. Therefore, a single oral inoculation could result in intestinal colonisation and rapid immune responses, removing the need for repeat dosing [72]. Consequently, several other live attenuated OCVs are currently in development.

*HaitiV*™, a new live attenuated OCV, has been developed from an O1 El Tor Ogawa *V. cholerae* variant isolated during the 2010 Haiti outbreak. It confers rapid protection and provides long-lasting immune protection from cholera in animal models [94,95]. Three further candidates that have demonstrated safety and immunogenicity in Phase I and II trials include Peru-15, based on O1 El Tor Inaba (C6709) recA deletion and nonmotile; Cuban 638, based on O1 El Tor Ogawa (C7258); and VA 1.4, based on non-toxigenic O1 El Tor Inaba [72].

Conventional vaccinology presents challenges when developing new vaccines and can be costly, time consuming, and labour intensive. Novel methods involving bioinformatic, subtractive proteomic, and immunoinformatic approaches to quickly prioritise potential vaccine targets and identify promising antigenic epitopes may play an important role in future cholera vaccine development [96]. Additionally, non-traditional antimicrobial strategies, including engineered nanoparticles that bind to and block key host receptors for cholera toxin, have shown promise in neutralising the effects of cholera toxin before it causes disease manifestations [97].

## 6. Unmet Needs in Cholera Vaccination

While new and novel approaches to cholera vaccine development must be welcomed, it is important to keep in mind the unmet needs that exist in the current landscape of cholera vaccination. This will help to ensure that research and resources are directed towards helping those most in need.

The protection of young children from cholera via vaccination is an area in which more progress is required. In young children, the risk of harm derives from insufficient protection following vaccination. A recent meta-analysis of oral cholera protection showed that children younger than 5 years are significantly less protected with a two-dose vaccinating regimen than those aged 5 years or older [98]. A separate study also found that children under 5 had minimal protection from a single-dose schedule over a 2-year period [99]. There is a need for new vaccine adjuvants, alternative dosing schedules, or new vaccines capable of eliciting long-lasting protection in young children, considering that current dosing regimens provide inadequate protection.

A further population with unmet needs is pregnant women. Here, rather than insufficient protection from vaccination, the challenge is insufficient data on the safety of cholera vaccination during pregnancy. Cholera in pregnancy has long been associated with high rates of stillbirths and miscarriages, with the severity of dehydration identified as the main risk factor for foetal death [100]. However, safety data for OCVs in pregnant women are controversial [101]. A recent meta-analysis of studies in pregnant women compared the effect of oral cholera vaccination on pregnancy outcomes, including adverse pregnancy outcome, miscarriage, stillbirth, preterm delivery, low birth weight, abortion, and malformation, with outcomes in unvaccinated women. This study showed no evidence of a relationship between oral cholera vaccination and adverse pregnancy outcomes, although the authors could not completely exclude the possibility that a vaccination regimen in pregnant women was completely risk-free, and further investigation is required [101].

## 7. Conclusions

Cholera is endemic in approximately 50 countries [8,11], primarily in South and Southeast Asia and in Africa [8,12]. In these areas, it remains a disease associated with poverty, causing illness and death in the poorest and most vulnerable people [102]. In developed nations, cholera is rare, and observed cases are almost always imported from areas where it is endemic by returning travellers [8,13,15]. These groups may include tourists, business travellers, those engaging in humanitarian, medical, or missionary work, or military personnel. Travellers may be at higher risk if they are visiting family and friends in an outbreak area or working in high-risk settings. Extended stays and reduced access to safe food and water increase the risk [8]; however, overall, cholera is a rare disease among travellers [15].

Irrespective of the setting, every case of cholera is preventable with the tools available to modern medicine. In developing nations, the WHO approach involves early detection and a quick response to contain outbreaks, coupled with effective mechanisms of coordination for technical support, resource mobilisation, and partnership at local and global levels [102]. The strategy recommends focussing on the relatively small areas most heavily affected by cholera, which experience cases on an ongoing or seasonal basis. In these areas, cholera transmission can be prevented through measures including improved water, sanitation, and hygiene services and the use of OCVs [102]. For international travellers, risk can be effectively mitigated by practicing regular hand hygiene and consuming food and water only from safe, known sources [27]. OCVs should be considered for travellers at high risk, who are likely to be directly exposed to cholera patients or contaminated water and food, particularly those staying in areas with limited access to healthcare facilities [27].

There are currently three WHO pre-qualified OCVs [61,62,63], which are based on killed whole-cell strains of both O1 and O139 serogroups. These vaccines offer significant protection in adults and children for up to 2 years, as demonstrated in multiple field trials. A fourth, live attenuated vaccine, previously licensed only in the USA until recently [65], has recently been approved by the European Medicines Agency for use in adults and children aged 6 years and older [67].

Live attenuated OCVs may mimic the natural infection process of *V. cholerae* more closely, and a single oral inoculation could result in intestinal colonisation and rapid immune responses, removing the need for repeat dosing [72]. These potential benefits have prompted the ongoing development of several additional live attenuated vaccines, although the safety of live vaccines with regard to reversion to an infective strain and spread within the local environment requires further investigation.

## Figures and Tables

**Table 1:** Summary table of recommendations for travellers from the USA, Canada, Australia, and key European countries.

Country	Recommending Bodies	Cholera Vaccination a Consideration for These Populations and under These Conditions
**Spain** 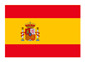	**Asociación Española de Vacunología (AEV)** [28,29]	ALL
	**Viajarseguro.org** [30,31]	NGO HCPs VFR
	**Ministerio de Sanidad** [32]	NGO HCPs VFR
**Italy** 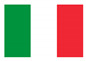	**Viaggiare Sicuri** [33]	NGO
	**Società Italiana di Medicina dei Viaggi e delle Migrazioni (SIMVIM) [34]**	No general advice—check website for specific recommendations according to type of trip
	**Ministero della Salute** [35]	ALL in endemic areas or during epidemic
**Germany** 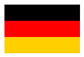	**Fit for Travel Germany** [36]	NGO HCPs VFR during epidemic or prolonged stay
	**Centrum für Reisemedizin (CRM)** [37]	NGO HCPs
	**Robert Koch Institute (RKI)** [38]	NGO HCPs VFR
**Switzerland** 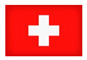	**Safetravel** [39]	NGO HCPs VFR
	**Bundesamt für gesundheit (BAG)** [40]	NGO SEA
	**Tropimed Suisse** [41]	NGO HCPs
	**Infovac** [42]	NGO HCPs SEA
**Sweden** 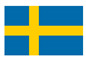	**1177 Vardguiden** [43]	VFR ALL during epidemic
	**Vaccin.se** [44]	NGO VFR
	**VaccinationsGuiden.se** [45]	ILL ALL in endemic areas or during epidemic
	**Public Health Agency (PHA)** [46]	NGO VFR ILL
**France** 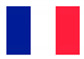	**Institut Pasteur de Lille** [47,48]	NGO
	**Institut Pasteur (Paris)** [49]	NGO HCPs
	**MesVaccins.net** [50]	NGO and HCPs only during an epidemic
**United Kingdom** 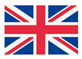	**NHS Fit for Travel [51]**	NGO HCPs VFR during epidemic
**Canada** 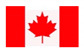	**Institut National de Santé Publique du Québec (INSPQ)** [52]	NGO HCPs VFR during epidemic ILL
**USA** 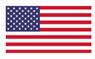	**Centers for Disease Control and Prevention (CDC);** **Advisory Committee on Immunization Practices** [53]	ALL adults (aged 18–64 years) during epidemic
**Australia** 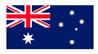	**Healthdirect.gov.au** [54]	NGO HCPs VFR ILL

ALL—all travellers who meet indication for cholera vaccination; HCPs—healthcare professionals working in at-risk environments (i.e., with refugees or during humanitarian disasters); ILL—any travellers at increased risk from cholera owing to existing medical conditions (i.e., bowel disease, diabetes, heart or kidney disease) or who are receiving certain medications (i.e., proton pump inhibitors); NGO—aid workers in at-risk environments (i.e., with refugees or during humanitarian disasters); SEA—travellers working on certain sea voyages; VFR—travellers visiting friends or relatives, or those who will have close contact with local populations.
